# Hepatic 3D spheroid models for the detection and study of compounds with cholestatic liability

**DOI:** 10.1038/srep35434

**Published:** 2016-10-19

**Authors:** Delilah F. G. Hendriks, Lisa Fredriksson Puigvert, Simon Messner, Wolfgang Mortiz, Magnus Ingelman-Sundberg

**Affiliations:** 1 Department of Physiology and Pharmacology, Section of Pharmacogenetics, Karolinska Institutet, Stockholm, Sweden; 2 InSphero AG, Schlieren, Canton of Zürich, Switzerland

## Abstract

Drug-induced cholestasis (DIC) is poorly understood and its preclinical prediction is mainly limited to assessing the compound’s potential to inhibit the bile salt export pump (BSEP). Here, we evaluated two 3D spheroid models, one from primary human hepatocytes (PHH) and one from HepaRG cells, for the detection of compounds with cholestatic liability. By repeatedly co-exposing both models to a set of compounds with different mechanisms of hepatotoxicity and a non-toxic concentrated bile acid (BA) mixture for 8 days we observed a selective synergistic toxicity of compounds known to cause cholestatic or mixed cholestatic/hepatocellular toxicity and the BA mixture compared to exposure to the compounds alone, a phenomenon that was more pronounced after extending the exposure time to 14 days. In contrast, no such synergism was observed after both 8 and 14 days of exposure to the BA mixture for compounds that cause non-cholestatic hepatotoxicity. Mechanisms behind the toxicity of the cholestatic compound chlorpromazine were accurately detected in both spheroid models, including intracellular BA accumulation, inhibition of *ABCB11* expression and disruption of the F-actin cytoskeleton. Furthermore, the observed synergistic toxicity of chlorpromazine and BA was associated with increased oxidative stress and modulation of death receptor signalling. Combined, our results demonstrate that the hepatic spheroid models presented here can be used to detect and study compounds with cholestatic liability.

Drug-induced liver injury (DILI) represents a serious problem for patient safety and is, together with drug-induced cardiac toxicity, one of the most common reasons for denial of drug approval and withdrawal of marketed drugs^1^. Cholestatic and mixed hepatocellular/cholestatic injuries constitute two major subtypes of DILI and may account for up to 50% of all DILI cases^2^. A notable example is the case of troglitazone, which was withdrawn from the market after reports of fulminant hepatic failure, for which later evidence was provided that the major metabolite troglitazone sulfate and to a lesser extent the parent drug troglitazone could pose cholestatic toxicity by interference with hepatobiliary transport and inhibition of the bile salt export pump (BSEP), thereby potentially contributing to troglitazone-induced liver injuries in humans^3^,^4^.

Drug-induced cholestasis (DIC) is primarily associated with impaired bile acid (BA) homeostasis, leading to the intrahepatic retention and accumulation of toxic BAs^5^. Hydrophobic BAs are particularly hepatotoxic and induce apoptosis via activation of death receptors^6^. DIC is often thought to result from interference of drugs or their metabolites with the function of BSEP, which is the predominant mediator of BA transport across the canalicular membrane, the rate-limiting step in bile formation^7^. Preclinical prediction of DIC therefore predominantly relies on assessing the potential of compounds to inhibit BSEP activity using membrane vesicles^8^ or hepatocytes in sandwich culture^9^. Although valuable, it is becoming increasingly apparent that a plethora of other mediators of BA homeostasis that play a role in cholestatic liver injury should be taken into consideration, including enzymes involved in BA conjugation and sulfation^10^, nuclear receptors^11^ and a variety of BA transporters^12^. Furthermore, symptoms of DIC *in vivo* may only appear weeks or months after starting treatment^13^, stressing the need for evaluation of the cholestatic risk of compounds upon long-term, repeated exposure.

A major limitation of the currently used *in vitro* models to predict adverse hepatic drug reactions such as cholestatic toxicity is the inability to maintain hepatic cells in a differentiated state. In simple 2D monolayer cultures, primary human hepatocytes (PHH) rapidly lose their phenotype due to dedifferentiation^14^, restricting their use to simple, acute toxicity studies. In sandwich culture, PHH form functional bile canalicular networks over the course of several days, which is of great value for studies of hepatobiliary transport and DIC^15^. Yet, sandwich-cultured PHH still gradually dedifferentiate over time, as evidenced by the presence of typical markers of epithelial-to-mesenchymal transition (EMT) after 2 weeks of culture^16^, which limits their use in assessing the chronic toxicity of compounds.

Cultivation of hepatic cells in 3D configuration as spheroids has been shown to better preserve the mature hepatocyte phenotype during long-term cultivation, because of the extensive formation of cell-cell contacts, reestablishment of cell polarity and production of extracellular matrices^17^. In 3D spheroid cultures, PHH closely resemble the liver *in vivo* on the proteome level^18^ and have functional bile canaliculi and stable liver-specific functionalities including albumin secretion and CYP activity for at least 5 weeks of culture^18^,^19^,^20^. We also recently provided proof of principle that PHH spheroids enable performing chronic toxicity studies and are suitable to study a variety of drug-induced liver injuries, including cholestasis, according to preliminary data^18^.

Liver cell lines overcome certain limitations met by PHH such as the high costs, scarcity and inter-donor variability, but are generally hampered by their immature phenotype. The HepaRG cell line bears several phenotypic characteristics of PHH^21^ and is unique in that it possesses functional bile canalicular networks with activity of hepatobiliary transporters comparable to PHH^22^. Accordingly, several studies have shown the suitability of 2D HepaRG cultures to study DIC^23^,^24^,^25^. When maintained in 3D spheroid culture, HepaRG cells have improved and stable functionality for several weeks of culture and in some cases their responsiveness to drug toxicity is improved^26^. To date, evaluation of the value of HepaRG 3D spheroid culture for studies of hepatobiliary drug transport and DIC is awaited.

It is clear that there is a need for novel *in vitro* assays to comprehensively evaluate the (chronic) cholestatic risk of compounds using a differentiated hepatic system. The aims of this study were to evaluate the suitability of two hepatic 3D spheroid models, one from PHH and one from HepaRG cells, to (i) detect compounds with cholestatic liability with emphasis on the importance of long-term, repeated exposures, and to (ii) recapitulate and identify the underlying mechanisms of DIC.

## Results

### PHH and HepaRG spheroids express relevant bile acid transporters

**Figure 1:**
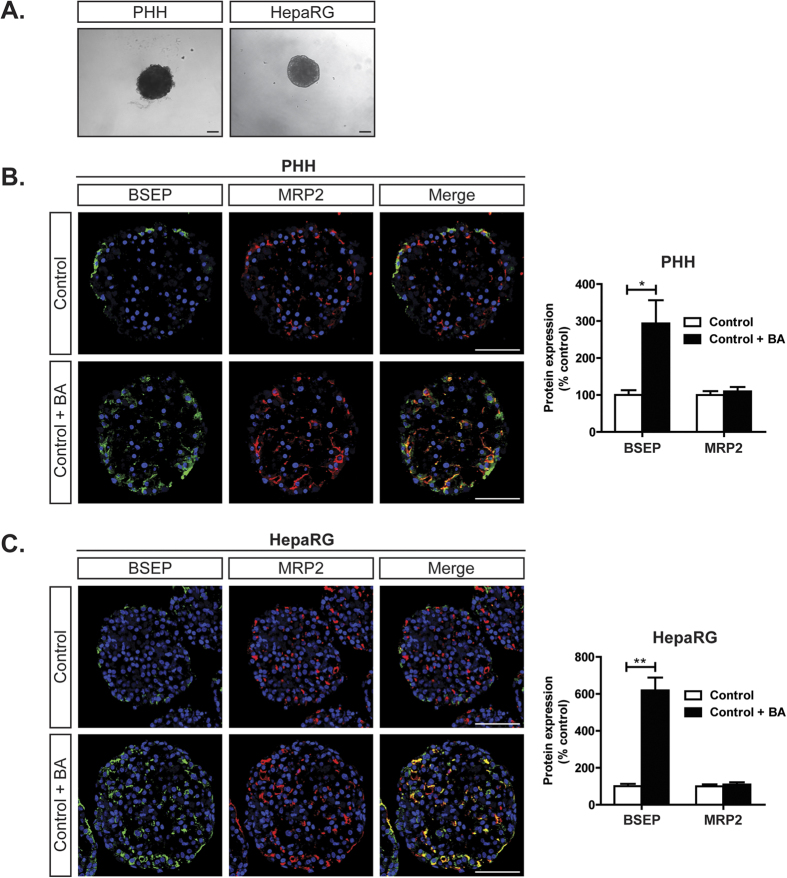
Characterization of PHH and HepaRG spheroids. (**A**) Morphology of PHH and HepaRG spheroids. (**B**,**C**) Immunohistochemical analysis for MRP2 and BSEP protein expression in PHH and HepaRG spheroids at day 16 and 13 respectively with or without 8 day exposure to a mixture of BAs. Quantification of the surface expression of the BA transporters was performed using CellProfiler software. *p < 0.05. All scale bars = 100 μm.

In the present study we evaluated two hepatic spheroid systems, one from PHH and one from HepaRG cells, as models for the detection and study of compounds with cholestatic liability. PHH and HepaRG cells aggregated into compact spheroids with defined borders after 7 and 4 days respectively and had a size of ~200 μm (Fig. 1A). We then evaluated the expression of two key BA transporters, multidrug resistance-associated protein 2 (MRP2) and bile salt export pump (BSEP). Both PHH and HepaRG spheroids expressed MRP2 abundantly throughout the spheroid. BSEP was exclusively expressed on the periphery, but was inducible upon exposure to a mixture of BAs (Fig. 1B,C; Table 1).

### Compounds with known cholestatic liability and bile acids pose synergistic toxicity in PHH and HepaRG spheroids

Hepatic 3D spheroids represent liver-like systems, enable long-term, repeated toxicity studies and express relevant BA transporters. Therefore, we studied the suitability of the PHH and HepaRG spheroid systems to identify compounds with cholestatic liability. Since it was recently shown that compounds with known cholestatic liability and an externally added mixture of BAs pose selective synergistic toxicity in sandwich-cultured hepatocytes^27^,^28^, we employed the strategy of using compound and BA co-exposures to identify compounds with cholestatic risk.

The BA mixture used in this study contained the six most abundant BAs found in human plasma (Table 1). The toxicity of the BA mixture was titrated to ensure that non-toxic concentrations were used for the co-exposures (Supplementary Fig. S1). We used a panel of compounds known to cause cholestatic or mixed cholestatic/hepatocellular injury (bosentan, chlorpromazine and troglitazone) and compounds known to cause non-cholestatic hepatotoxicity (acetaminophen and tetracycline) to validate the models. To classify the cholestatic risk of the tested compounds, we introduced the cholestatic index (CIx), which is a measure of the extent of interference with the extrusion of the added BAs posed by the compound and is defined as the ratio between the EC_50_-value resulting from compound and BA co-exposure and the EC_50_-value resulting from exposure to the same compound alone. Compounds were deemed to possess cholestatic risk when CIx ≤ 0.80.

**Figure 2:**
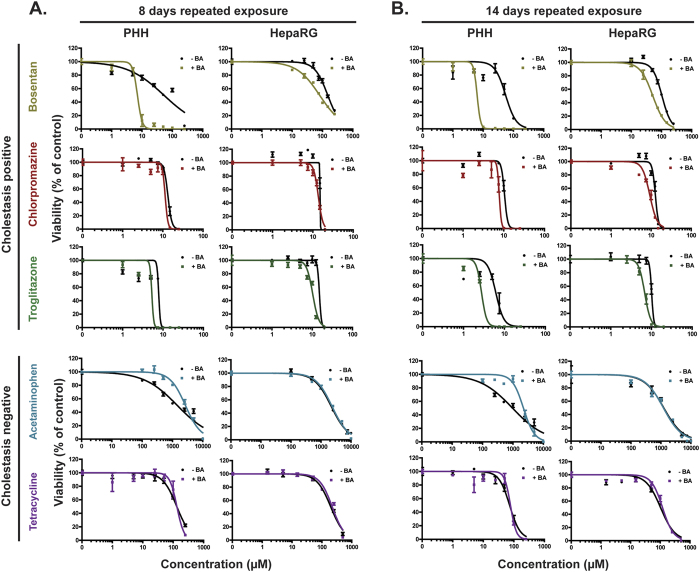
Exposure to hepatotoxins in the presence of bile acids reveals a selective synergistic toxicity of compounds with known cholestatic liability and bile acids. PHH and HepaRG spheroids were repeatedly exposed to a set of compounds with known cholestatic liability (bosentan, chlorpromazine and troglitazone) and compounds with non-cholestatic hepatotoxicity patterns (acetaminophen and tetracycline) in the presence or absence of a non-toxic BA mixture for 8 days (**A**) and 14 days (**B**). Cellular ATP content was measured as a marker for viability. Each data point is the mean ± SEM viability of n = 4–6 spheroids. Data are representative of 3 independent experiments.

**Figure 3:**
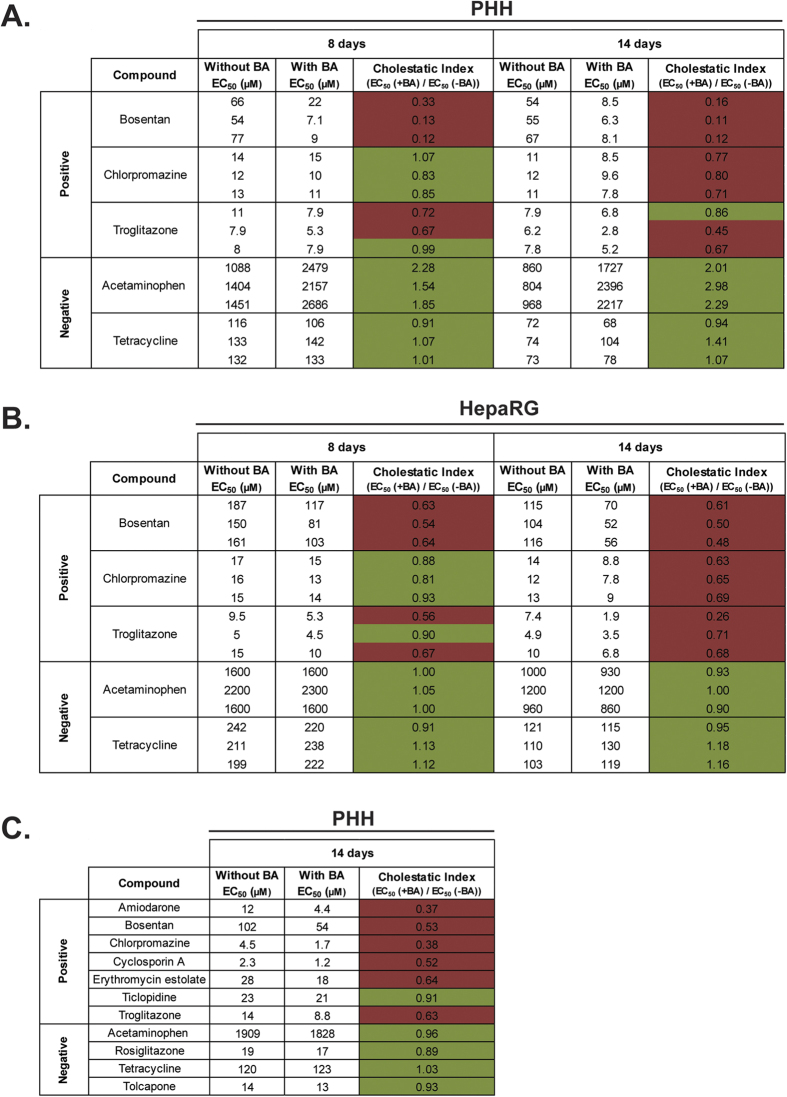
Cholestatic risk classification of the tested hepatotoxins based on the cholestatic index (CIx). (**A,B**) Classification of the cholestatic risk of the tested compounds in PHH and HepaRG spheroids after 8 and 14 days of repeated exposure as determined by the cholestatic index (CIx). Three independent experiments for each compound are presented. Viability was assessed by measuring the cellular ATP content. (**C**) CIx values of a panel of hepatotoxins determined in multi-donor PHH spheroids after 14 days of repeated exposure. Viability was assessed by measuring albumin secretion, which has similar sensitivity to measuring the cellular ATP content in terms of determining the CIx values (Supplementary Fig. S2). For all figures: green: CIx ≥ 0.80; red: CIx ≤ 0.80.

In PHH spheroids, co-exposure to bosentan and BAs for 8 days resulted in increased toxicity in a dose-dependent manner compared to bosentan exposure alone (CIx of 0.19 ± 0.12) (Figs 2A and 3A). Similarly, an increase in toxicity upon co-exposure to troglitazone and BAs was observed compared to troglitazone exposure alone (CIx = 0.80 ± 0.17). A slight increase in toxicity upon co-exposure to chlorpromazine and BAs was observed, but based on the CIx value chlorpromazine was classified as a compound with low cholestatic risk (CIx = 0.90 ± 0.12). In contrast, we observed no synergistic toxicity of BAs and the non-cholestatic hepatotoxins acetaminophen (CIx = 1.89 ± 0.37) or tetracycline (CIx = 1.00 ± 0.08). Rather, a consistent protective effect of the added BAs was observed at normally toxic concentrations of acetaminophen (Figs 2A and 3A).

In HepaRG spheroids, co-exposure to bosentan and BAs resulted in an increase in toxicity compared to exposure to bosentan alone (CIx = 0.60 ± 0.06). Likewise, co-exposure to BAs and either troglitazone or chlorpromazine resulted in enhanced toxicity compared to exposure to the respective compound alone (Figs 2A and 3B). Based on the CIx values, troglitazone (CIx = 0.71 ± 0.17) and chlorpromazine (CIx = 0.87 ± 0.06) were classified as compounds with cholestatic and low cholestatic risk, respectively. Similar to in PHH spheroids, acetaminophen (CIx = 1.02 ± 0.03) and tetracycline (CIx = 1.05 ± 0.12) were classified as compounds with low cholestatic risk (Figs 2A and 3B).

### Prolonged exposure increases the synergistic toxicity of compounds with cholestatic liability and bile acids

Next, we assessed whether prolonging the drug and BA co-exposures from 8 to 14 days would further improve the cholestatic risk classification of the tested compounds. Regardless of the presence of the BA mixture, both PHH and HepaRG spheroids were more sensitive to the toxicity of all hepatotoxins after 14 days compared to 8 day exposure (compare Fig. 2A,B). Importantly, the synergistic toxicity of the tested compounds with known cholestatic liability and BAs was more pronounced after 14 days of exposure, seen by the reduced CIx values (Fig. 3A,B). Most notably, the synergistic toxicity of chlorpromazine and BAs was exacerbated, where chlorpromazine was classified as a compound with cholestatic risk in both PHH spheroids (CIx = 0.78 ± 0.05) and HepaRG spheroids (CIx = 0.66 ± 0.03) exclusively after 14 days of repeated exposure. The non-cholestatic hepatotoxins acetaminophen and tetracycline remained classified as compounds with low cholestatic risk in both spheroid models, where no increase in toxicity was observed for both compounds in the presence of the BA mixture (Figs 2B and 3A,B).

### Cholestatic risk classification of a panel of hepatotoxins in multi-donor PHH spheroids

To further validate our findings, we tested an extended set of hepatotoxins using spheroids from PHH of 10 individual donors. After 14 days of repeated exposure, compounds known to cause cholestatic or mixed cholestatic/hepatocellular injury (amiodarone, bosentan, chlorpromazine, cyclosporin A, erythromycin estolate and troglitazone) were flagged as compounds having cholestatic risk, with the exception of ticlopidine (CIx = 0.91; low cholestatic risk). The tested non-cholestatic hepatotoxins (acetaminophen, rosiglitazone, tetracycline and tolcapone) were classified as compounds with low cholestatic risk (Fig. 3C).

### Cholestatic patterns of chlorpromazine-induced toxicity are recapitulated in PHH and HepaRG spheroids

**Figure 4:**
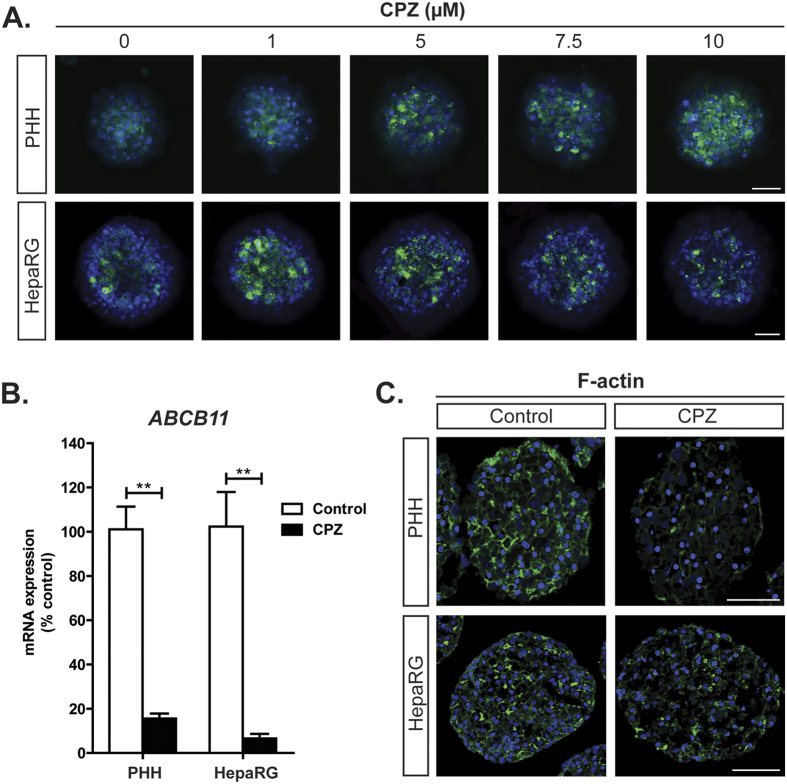
Mechanisms of chlorpromazine-induced cholestasis are recapitulated in PHH and HepaRG spheroids. PHH and HepaRG spheroids were repeatedly exposed to the model cholestatic compound chlorpromazine (CPZ) for 8 days. (**A**) BA accumulation was assessed by using the fluorescently labelled taurocholic acid derivative tauro-nor-THCA-24-DBD. Note that 10 μM CPZ was toxic to the HepaRG spheroids. (**B**) Expression of *ABCB11*, encoding BSEP, was analysed by RT-qPCR and normalized to the expression of the housekeeping gene *GAPDH*. Data represent the means ± SEM of 3 independent experiments. **p < 0.01. (**C**) F-actin cytoskeleton integrity was visualized by Phalloidin staining. All scale bars = 100 μm.

Next, we evaluated whether the PHH and HepaRG spheroid models can recapitulate the mechanisms associated with DIC. To this end, we used the model cholestatic compound chlorpromazine and monitored its effect on intracellular BA accumulation. Repeated exposure to chlorpromazine for 8 days resulted in the cytoplasmic accumulation of the fluorescently labelled taurocholic acid derivative tauro-nor-THCA-24-DBD (Fig. 4A). We previously reported that chlorpromazine inhibited the expression of *ABCB11*, encoding BSEP, in PHH spheroid culture^18^. Here, we confirmed this finding in both PHH and HepaRG spheroids (Fig. 4B). Chlorpromazine-induced cholestasis has furthermore been associated with the disruption of the F-actin cytoskeleton distribution and integrity in 2D HepaRG culture^23^. Consistently, chlorpromazine also reduced F-actin expression and disrupted its integrity in both PHH and HepaRG spheroids (Fig. 4C).

### The synergistic toxicity of chlorpromazine and bile acids is associated with increased oxidative stress

Chlorpromazine and BAs are both independently known to induce oxidative stress^23^,^29^ and hence we wondered whether an increase in oxidative stress could explain the observed synergistic toxicity of chlorpromazine and BAs. To this end, we studied the expression profiles of PHH and HepaRG spheroids after exposure to chlorpromazine in the presence or absence of BAs, where only the co-exposure condition caused noticeable toxicity.

**Figure 5:**
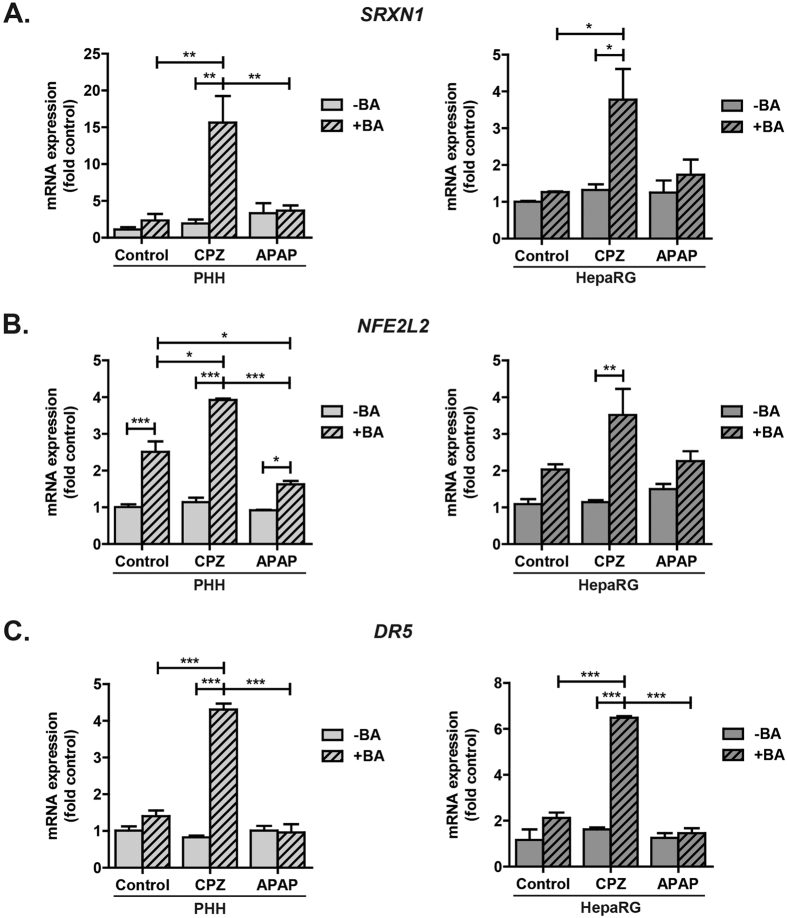
Oxidative stress and modulation of death receptor signalling underlie the synergistic toxicity of chlorpromazine and bile acids. PHH and HepaRG spheroids were repeatedly exposed to chlorpromazine (CPZ) or acetaminophen (APAP) in the presence or absence of a non-toxic BA mixture. After 8 days, expression levels of the Nrf2 target gene *SRXN1* (**A**), *NFE2L2* (**B**) and *DR5* (**C**) were evaluated by RT-qPCR and normalized to the expression of the housekeeping gene *GAPDH*. Data represent the means ± SEM of 3 independent experiments. *p < 0.05, **p < 0.01, ***p < 0.001.

First, we evaluated the expression levels of sulfiredoxin 1 (*SRXN1*), the direct target of nuclear factor (erythroid-derived 2)-like 2 (Nrf2)^30^. In both spheroid models, co-exposure to chlorpromazine and BAs resulted in significant induction of *SRXN1* compared to exposure to either chlorpromazine or BAs alone (Fig. 5A). Analysis of expression of *NFE2L2*, encoding Nrf2, revealed that BAs readily induced *NFE2L2* and that co-exposure to chlorpromazine and BAs significantly further increased *NFE2L2* expression (Fig. 5B).

Interestingly, analysis of the expression profiles of PHH and HepaRG spheroids exposed to the non-cholestatic hepatotoxin acetaminophen, at a concentration that was sub-toxic both in the presence or absence of BAs, revealed no changes in *SRXN1* or *NFE2L2* expression upon co-exposure to acetaminophen and BAs compared to exposure to acetaminophen or BAs alone (Fig. 5A,B), suggesting that a selective cholestatic mechanism accounts for the synergistic increase in *SRXN1* and *NFE2L2* expression observed upon co-exposure to chlorpromazine and BAs.

### Chlorpromazine-induced accumulated toxic bile acids modulate death receptor signalling

Toxic BAs have been reported to induce death receptor 5 (*DR5*) and its subsequent aggregation, leading to the sensitization of hepatocytes to death receptor-mediated apoptosis^31^. Therefore, we wondered whether the synergistic toxicity of chlorpromazine and BAs could be explained by chlorpromazine-induced accumulation of toxic BAs, which subsequently modulate death receptor signalling.

Co-exposure to chlorpromazine and BAs significantly induced *DR5* compared to exposure to either chlorpromazine or BAs alone in both PHH and HepaRG spheroids (Fig. 5C). Interestingly, exposure to a toxic concentration of BAs also resulted in induction of *DR5*, whereas no change in *DR5* expression was observed after exposure to a toxic concentration of chlorpromazine alone (Supplementary Fig. S3). In addition, no induction of *DR5* was observed upon co-exposure to the non-cholestatic hepatotoxin acetaminophen and BAs, which is likely due to the lack of accumulation of toxic BAs (Fig. 5C). Taken together, these findings provide evidence that the induction of *DR5* seen after co-exposure to chlorpromazine and BAs is indeed likely driven by the accumulated toxic BAs caused by chlorpromazine.

## Discussion

Drug-induced cholestasis (DIC) is an important clinical problem and causes withdrawal of drugs in development. Preclinical prediction of DIC is currently mainly limited to measuring the compound’s potential to inhibit BSEP. However, manifestation of DIC is often complex, multifactorial and delayed in onset^13^,^32^,^33^. Thus, there is a need for novel *in vitro* assays that holistically evaluate the cholestatic risk of compounds in a physiologically relevant liver model that is applicable to chronic toxicity studies.

Here, we evaluated two hepatic 3D spheroid systems, one from PHH and one from HepaRG cells, for the detection and study of compounds with cholestatic liability. We first characterized the models by confirming the protein expression of the two main apical BA transporters, MPR2 and BSEP, of which the expression of the latter was inducible by BAs (Fig. 1). Using repeated exposure for 8 days to a set of compounds known to cause cholestatic or mixed cholestatic/hepatocellular injury in the presence or absence of a non-toxic concentrated BA mixture, we observed synergistic toxicity of the added BAs and the compounds troglitazone and bosentan, and to a lesser extent with chlorpromazine, compared to exposure to the compounds alone (Figs 2A and 3). This toxic synergistic interaction is thought to result from interference with the extrusion of the added BAs posed by these compounds, resulting in the accumulation of intracellular BAs to toxic concentrations that promote cell death.

To classify the cholestatic risk of the tested compounds, we introduced the cholestatic index (CIx). After 8 days of repeated compound/BA co-exposures in both PHH and HepaRG spheroid systems bosentan and troglitazone were classified as compounds with cholestatic risk (CIx values ≤ 0.80), whereas chlorpromazine was classified as a compound with low cholestatic risk. The non-cholestatic hepatotoxins acetaminophen and tetracycline were, based on the CIx values, classified as compounds with low cholestatic risk. In contrast, a consistent protective effect of the added BAs was observed upon exposure to acetaminophen in PHH spheroids, but not in HepaRG spheroids (Figs 2A and 3A,B). Indeed, it has been described that BAs can have a critical protective role in the initiation and recovery of acetaminophen-induced liver injury in mice^34^.

Extending the compound/BA co-exposures from 8 to 14 days intensified the synergistic toxicity of the compounds with known cholestatic liability and the BA mixture in both PHH and HepaRG spheroid models resulting in improved cholestatic risk classification of the tested compounds (Figs 2B and 3A,B). Importantly, based on the CIx values, prolonging the exposures resulted in a shift of classification of chlorpromazine from having a low cholestatic risk after 8 days to having cholestatic risk after 14 days, thus emphasizing the value of performing long-term, repeated exposures. Compared to bosentan and troglitazone, a longer time of co-exposure to chlorpromazine and the BA mixture was needed before the cholestatic risk of chlorpromazine was identified in the hepatic spheroid systems. This could be caused by a proportionally higher toxicity mediated by other mechanisms not influenced by the external BAs after short-term exposure. In addition, chlorpromazine only moderately inhibits BSEP (IC_50_ = 147.6 μM), whereas troglitazone (IC_50_ = 2.7 μM) and bosentan (IC_50_ = 38.1 μM) more potently inhibit BSEP, which therefore might induce more direct accumulation of the external BAs resulting in the detection of the cholestatic effects of these compounds already after 8 days of exposure. Collectively, the PHH and HepaRG spheroid models performed very similarly in detecting the cholestatic liability of the tested compounds. Further investigations comparing both spheroid models are needed to make more definite conclusions about the competence of the HepaRG spheroid model.

A subsequent screen with a larger panel of hepatotoxins in multi-donor PHH spheroids correctly identified the cholestatic risk of all but one of the compounds (Fig. 3C). Ticlopidine, known to cause mixed hepatocellular/cholestatic injury, was in this PHH spheroid system classified as a compound with low cholestatic risk. Ticlopidine-induced cholestatic liver injury is not fully understood, but is often seen in an idiosyncratic manner. In rats, extensive secretion of glutathione conjugates of ticlopidine by MRP2 altered the bile composition and decreased the biliary secretion of phospholipids, which may contribute to the cholestatic hepatotoxicity of ticlopidine^35^. Furthermore, studies point towards a pivotal role of the adaptive immune system, since ticlopidine stimulated T cells of patients with ticlopidine-induced cholestatic hepatitis *in vitro*, but not T cells from healthy controls^36^. In addition, an association between severe ticlopidine-induced cholestatic liver injury and the human leukocyte antigen (HLA) allele HLA-A*3303 was reported in Japanese patients^37^. It is evident that the hepatic spheroid models presented here cannot detect idiosyncratic immune-mediated processes through drug-induced cholestatic toxicity. In general, no *in vitro* system currently exists that can detect idiosyncratic immune-mediated DILI reactions, due to the complexity of the immune system, which harbours many different cell types and requires interactions between parenchymal and non-parenchymal cells. Rather, the hepatic spheroid models serve as a tool to screen for the cholestatic liability of compounds by means of assessing the drug-induced interference with BA homeostasis, which can constitute a risk factor for the development of DILI. Using PHH spheroids from donors with specific polymorphisms, e.g. in *ABCB11* encoding BSEP, might constitute an opportunity to investigate patient-specific risk factors in the development of drug-induced cholestatic liver injuries.

Toxicity mechanisms of the model cholestatic compound chlorpromazine were accurately detected in both PHH and HepaRG spheroid systems. Chlorpromazine induced dose-dependent BA accumulation and inhibited expression of *ABCB11*, encoding BSEP, in both spheroid models (Fig. 4A,B). Although inhibition of *ABCB11* expression is thought to be a secondary effect in chlorpromazine-induced cholestasis^23^, a recent study reported that a number of potent BSEP inhibitory drugs also were potent repressors of *ABCB11* expression^38^, suggesting it to be an important contributing mechanism to DIC. PHH and HepaRG spheroids exposed to chlorpromazine also showed reduced F-actin expression and disruption of its integrity (Fig. 4C), which have been reported to constitute early events in chlorpromazine-induced cholestasis through the induction of oxidative stress^23^. The F-actin cytoskeleton is required for proper insertion and localization of BA transporters at the canalicular membrane and disruption of its integrity has been associated with intracellular BA accumulation due to the internalization of BA transporters^39^,^40^.

**Figure 6:**
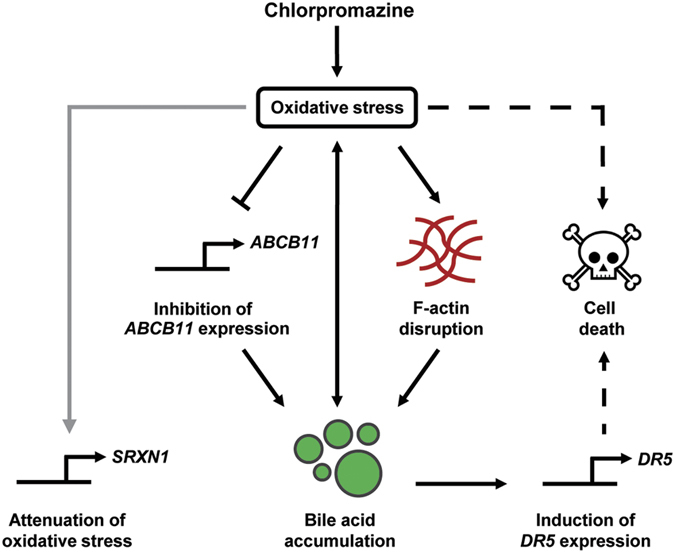
Working model for chlorpromazine-induced cholestatic toxicity. Chlorpromazine induces oxidative stress^23^, which is associated with the disruption of the F-actin cytoskeleton integrity, leading to intracellular BA accumulation. The oxidative stress is further synergistically enhanced by chlorpromazine and the accumulated BAs at toxic concentrations. In addition, chlorpromazine inhibits the expression of *ABCB11*, encoding BSEP, further aggravating BA accumulation. The accumulated toxic BAs induce *DR5* expression, leading to the activation of the death receptor signalling pathway. Collectively, these events result in cell death.

This study and other recent studies^27^,^28^ reported selective synergistic toxicity of compounds with cholestatic liability and BAs. The mechanisms underlying this synergism were hitherto unknown. Here, we associated the synergistic toxicity of chlorpromazine and BAs with increased oxidative stress, seen by the induction of the Nrf2 target *SRXN1* (Fig. 5A). Interestingly, *NFE2L2*, encoding Nrf2, itself was strongly induced upon chlorpromazine and BA co-exposure, and to a lesser extent upon BA exposure alone as previously described^41^ (Fig. 5B). BAs are known to activate Nrf2^42^, which in turn can induce hepatic *ABCC1–4*, encoding MRP1–4^43^. Therefore, we hypothesize that the observed *NFE2L2* induction constitutes a protective mechanism to reduce the intracellular BA concentration, since MRP1–4 are all implicated in transporting BAs into the blood or bile^44^. In addition to an increase in oxidative stress, we associated the synergistic toxicity of chlorpromazine and BAs with induction of *DR5*, most likely mediated by the accumulated toxic BAs (Fig. 5C). BAs at toxic concentrations are known to promote apoptosis by activation of the death receptor signalling pathway. Toxic BAs induce *DR5* through JNK activation^31^,^45^ and increase the transport of Fas to the cell surface^46^, which induces oligomerization of death receptors and formation of the death-inducing signalling complex (DISC), ultimately leading to hepatocyte apoptosis. Figure 6 summarizes the currently identified mechanisms of chlorpromazine-induced cholestasis. These mechanistic investigations further demonstrate the validity of the hepatic spheroid models to study drug-related cholestatic liabilities.

To date, various different models have been developed in order to assess the cholestatic liability of compounds. Assays employing membrane vesicles provide valuable information about drug-induced interference with efflux transporter activity, but this does not always correlate with the risk of a drug to induce cholestatic hepatotoxicity, exemplified by a number of compounds that are weak BSEP inhibitors but still induce cholestatic liver injury^47^. Recent promising developments using hepatocyte sandwich cultures have been made^27^,^28^, which allow a more comprehensive assessment of the cholestatic risk of compounds. Yet, hepatocyte sandwich cultures can be complex to handle, require high cell numbers and have low throughput, hampering their use in routine toxicity testing. Furthermore, drug exposures have generally been limited to 72 hours in hepatocyte sandwich cultures, questioning the ability of the culture system to assess chronic drug toxicity. The hepatic spheroid models used in this study require low cell numbers and allow a higher throughput, where screening is performed in 96-well format. Furthermore, especially with regard to the PHH spheroid model, the use of a well-differentiated system that closely mimics the *in vivo* liver and is able to maintain a mature phenotype for several weeks in culture^18^ is valuable, especially to assess chronic drug toxicity events.

In conclusion, we provide proof of principle that hepatic spheroid models from PHH or HepaRG cells can be used for the detection and study of compounds with cholestatic liability using long-term, repeated drug and BA co-exposures. The hepatic spheroid models presented here might aid in assessing the cholestatic liability of compounds during preclinical safety testing.

## Methods

### Materials

Cell culture medium, medium supplements, and compounds were obtained from Sigma-Aldrich (Sweden) or Life Technologies (Sweden) unless otherwise stated.

### Primary human hepatocyte spheroid cultures

Cryopreserved PHH (Bioreclamation IVT, USA) were thawed according to the supplier’s instructions. The cells were seeded in PHH medium (Williams’ medium E containing 100 units/ml penicillin, 100 μg/ml streptomycin, 2 mM L-glutamine, 10 μg/ml insulin, 5.5 μg/ml transferrin, 6.7 ng/ml sodium selenite, 100 nM dexamethasone) supplemented with 10% fetal bovine serum. The cells were seeded at a density of 1,500 viable cells per well in ultra-low attachment (ULA) plates (Corning). When PHH had reassembled into compact aggregates (day 4–6 after seeding), spheroids were shifted to serum-free PHH medium. PHH spheroids from cryopreserved multi-donor PHH were obtained from InSphero AG (MT-02-302-01, lot IPHH_11). The hepatocytes were derived from 5 male and 5 female donors and were aggregated into 3D microtissues in GravityPLUS™ hanging drop plates and cultivated and assayed in GravityTRAP™ plates. The spheroids were maintained in serum-free PHH medium.

### HepaRG spheroid cultures

Cryopreserved differentiated HepaRG cells (Biopredic International, France) were thawed according to the supplier’s instructions. The cells were seeded in Williams’ medium E with GlutaMAX^TM^ containing the thaw, seed and general purpose additive (ADD670; Biopredic International). The cells were seeded at a density of 2,000 viable cells per well in ULA plates. From day 5, the spheroids were maintained in medium containing 0.5% DMSO, the serum-free induction additive (ADD650, Biopredic International), 20 ng/ml epidermal growth factor and 10 ng/ml hepatocyte growth factor (HumaXpress, Nordic BioSite, Sweden).

### Bile acid mixture

A mixture of six BAs was used (Table 1). The stock was prepared in DMSO according to the relative concentration of each BA found in normal human plasma^48^.

### Selection of compounds

Compounds with different DILI patterns were screened in the hepatic spheroid systems. DILI categorizations for the following compounds were adapted from Gustafsson *et al*.^49^: Compounds with mixed hepatocellular/cholestatic toxicity patterns: amiodarone, erythromycin estolate, ticlopidine, troglitazone; compounds with cholestatic toxicity patterns: bosentan, chlorpromazine and cyclosporin A; and compounds with hepatocellular toxicity patterns: acetaminophen and tolcapone. Rosiglitazone and tetracycline were classified as compounds with patterns of hepatocellular and steatotic toxicity based on refs 50 and 51, respectively.

### Toxicity studies

Stock solutions of the compounds were diluted in culture medium to a maximal final DMSO concentration of 0.3% for PHH spheroids and 0.6% for HepaRG spheroids. Toxicity studies were started at day 8 and day 5 for PHH and HepaRG spheroids, respectively. Spheroids were treated every other day with the compounds in the presence or absence of the BA mixture. Cellular ATP content was determined as a marker of cell viability using the CellTiter-Glo Luminescent Cell Viability Assay (Promega, Sweden). When indicated, albumin concentrations were determined by ELISA (Bethyl laboratories, USA). EC_50_ values (the concentration of the compound causing a 50% reduction in cell viability) were determined using GraphPad Prism (GraphPad Software, USA).

### Cholestatic risk classification of compounds

To evaluate the cholestatic risk of the tested compounds we introduced the cholestatic index (CIx). The CIx is defined as the ratio between the EC_50_-value from co-exposure to a compound and the BA mixture and the EC_50_-value from exposure to the compound alone. Compounds with a CIx ≥ 0.80 (i.e. 20% or less difference in cell viability between compound and BA co-exposure and compound exposure alone) were considered to have a low cholestatic risk, whereas compounds with a CIx ≤ 0.80 were considered to have cholestatic risk.

### Bile acid accumulation

BA accumulation was assessed with a fluorescently labelled taurocholic acid derivative, tauro-nor-THCA-25-DBD (Genomembrane Company, Ltd, Japan). After 6 days of chlorpromazine exposure, the spheroids were co-exposed to chlorpromazine and 20 μM tauro-nor-THCA-25-DBD for an additional 2 days. Fluorescence was assessed by confocal microscopy (Zeiss LSM 710).

### Immunohistochemistry

Spheroids were fixed in 4% paraformaldehyde at 4˚C O/N. Immunohistochemistry was performed on cryosections (8 μm) for BSEP (Atlas, Sweden) and MRP2 (Abcam, UK). The donkey anti-rabbit Alexa Fluor 488 and donkey anti-mouse Alexa Fluor 555 were used as secondary antibodies. F-actin was visualized using Alexa Fluor 488 Phalloidin. Slides were mounted with ProLong Gold Antifade Mountant with DAPI and fluorescence was assessed by confocal microscopy (Zeiss LSM 710). Surface expression of the BA transporters was quantified using CellProfiler software.

### RT-qPCR analysis

Total RNA was extracted using Qiazol according to the manufacturer’s protocol. RNA was reverse-transcribed into cDNA with SuperScript III reverse transcriptase. RT-qPCR analysis was performed with a 7500 Fast Real-Time PCR system using a TaqMan Universal or SYBR Green mix.

### Statistics

One-way ANOVA with Bonferroni’s post hoc test or student’s *t*-test was applied to compare data between treated and control spheroids.

## Additional Information

**How to cite this article**: Hendriks, D. F. G. *et al*. Hepatic 3D spheroid models for the detection and study of compounds with cholestatic liability. *Sci. Rep.* **6**, 35434; doi: 10.1038/srep35434 (2016).

## Supplementary Material

Supplementary Information

## Figures and Tables

**Table 1:** Composition of the bile acid mixture and the corresponding concentrations found in human plasma.

Bile acid	Concentration in human plasma (μM)^48^
Cholic acid	0.41
Chenodeoxycholic acid	0.64
Deoxycholic acid	0.48
Lithocholic acid	0.008
Ursodeoxycholic acid	0.14
Glycochenodeoxycholic acid	0.80
Sum	2.478
