# Supplementary material for: Hepatic 3D spheroid models for the detection and study of compounds with cholestatic liability
Source: Sci Rep. 2016 Oct 19;6:35434. doi: 10.1038/srep35434 (PMC5069690; doi:10.1038/srep35434)
Supplement: Supplementary Information [file srep35434-s1.pdf]

Hepatic 3D spheroid models for the detection and study of compounds with cholestatic liability

Delilah F. G. Hendriks[1*], Lisa Fredriksson Puigvert[1*], Simon Messner[2], Wolfgang Mortiz[2], Magnus Ingelman-Sundberg[1]

[1] Department of Physiology and Pharmacology, Section of Pharmacogenetics, Karolinska Institutet, Stockholm, Sweden

[2] InSphero AG, Schlieren, Canton of Zürich, Switzerland

[§] These authors contributed equally to this work.

Delilah F. G. Hendriks, Karolinska Institutet, Department of Physiology and Pharmacology, Section of Pharmacogenetics, Nanna Svartz Väg 2, 17177 Stockholm, Sweden, Phone: +46 8524 877 11, Fax: +46 8337 327, E-mail: delilah.hendriks@ki.se

Lisa Fredriksson Puigvert, Karolinska Institutet, Department of Physiology and Pharmacology, Section of Pharmacogenetics, Nanna Svartz Väg 2, 17177 Stockholm, Sweden, Phone: +46 8524 877 62, Fax: +46 8337 327, E-mail: fredrikssonle@gmail.com

Simon Messner, InSphero AG, Schlieren, Canton of Zürich, Switzerland, Phone: +41 44 515 0494, Fax: +41 44 515 0491, E-mail: simon.messner@insphero.com

Wolfgang Moritz, InSphero AG, Schlieren, Canton of Zürich, Switzerland, Phone: +41 44 515 0501, Fax: +41 44 515 0491, E-mail: wolfgang.moritz@insphero.com

***** Corresponding author: Dr. Magnus Ingelman-Sundberg, Karolinska Institutet, Department of Physiology and Pharmacology, Section of Pharmacogenetics, Nanna Svartz Väg 2, 17177 Stockholm, Sweden, Phone: +46 8524 877 35, Fax: +46 8337 327, E-mail: Magnus.Ingelman-Sundberg@ki.se

Supplementary Information

Included Supplementary Material:

Supplementary Figures 1-3

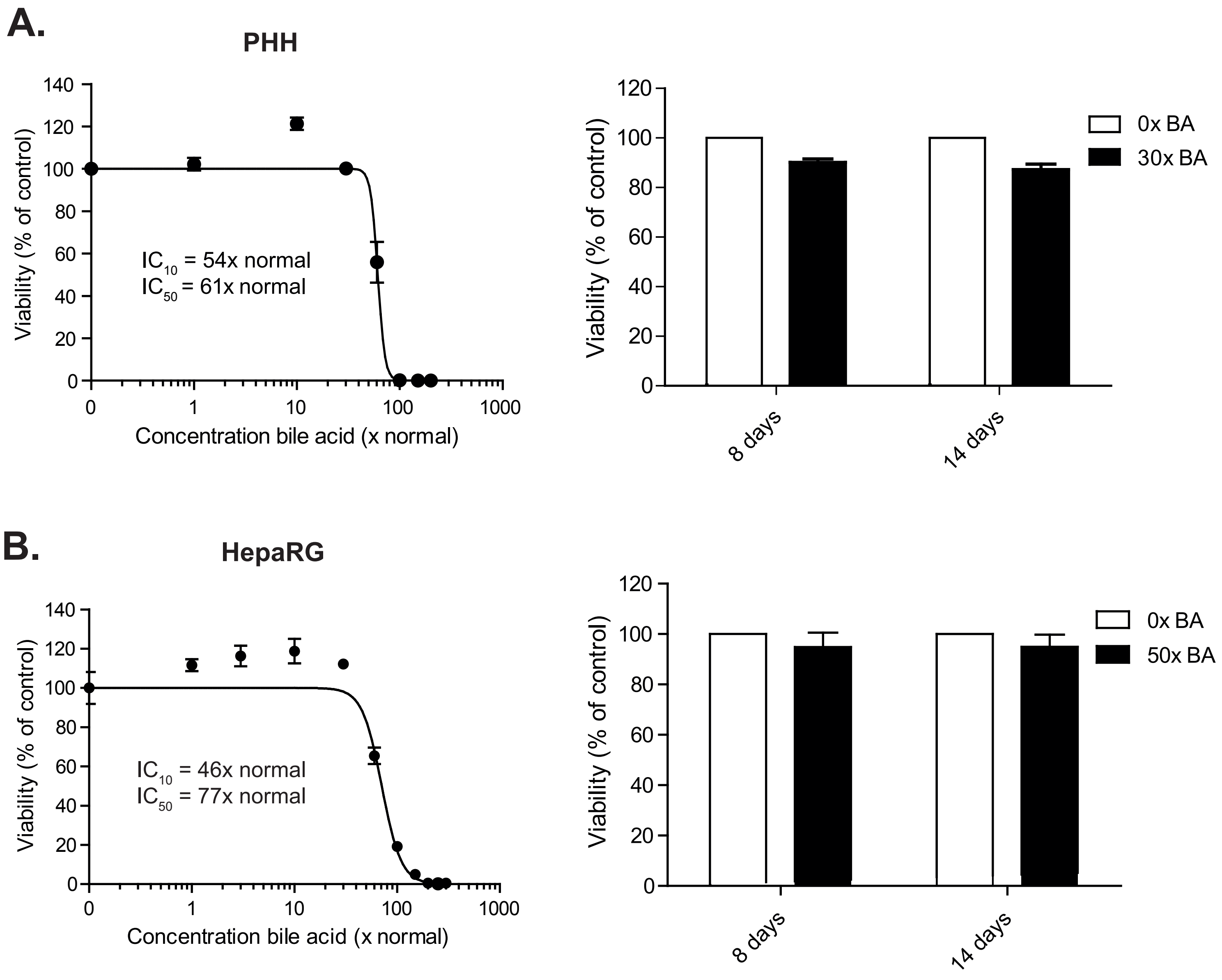

Supplementary figure S1. Titration of bile acid toxicity in PHH and HepaRG spheroids.

The toxicity of the BA mixture, concentrated according to the respective concentration of each BA in human plasma, was titrated in PHH spheroids (A) and HepaRG spheroids (B) after 8 days of repeated exposure. The maximal non-toxic concentration of the BA mixture was determined to be 30-50x for PHH and 50x for HepaRG spheroids, respectively. The BA mixture remained non-toxic after 14 days of repeated exposure.

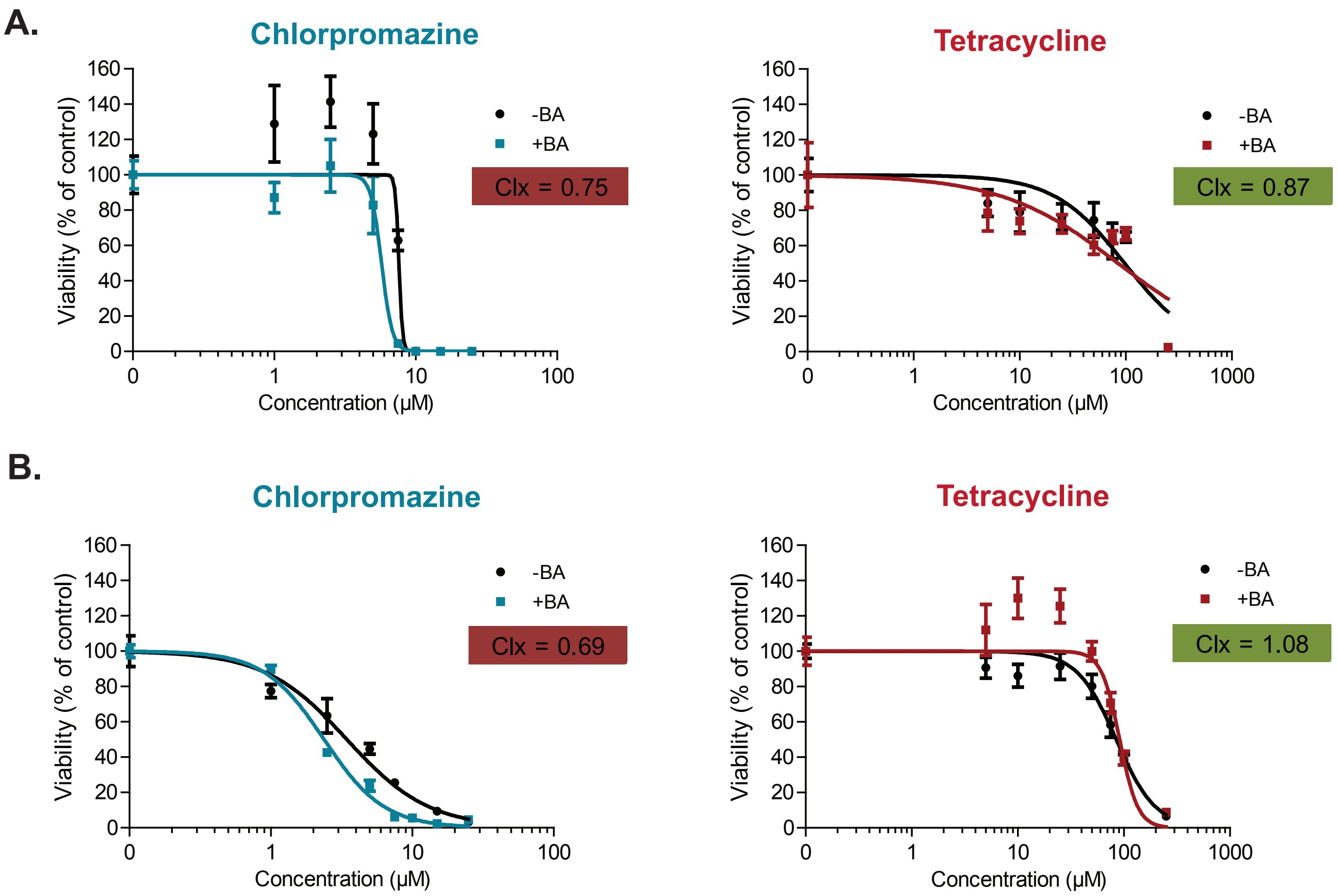

Supplementary figure S2. ATP and albumin as toxicity read-outs have similar sensitivity in determining the cholestatic risk of compounds.

PHH spheroids were repeatedly exposed to chlorpromazine or tetracycline for 8 days in the presence or absence of the BA mixture. Cholestatic risk classification of both compounds was compared using cellular ATP content (A) or albumin secretion (B) as toxicity read-outs.

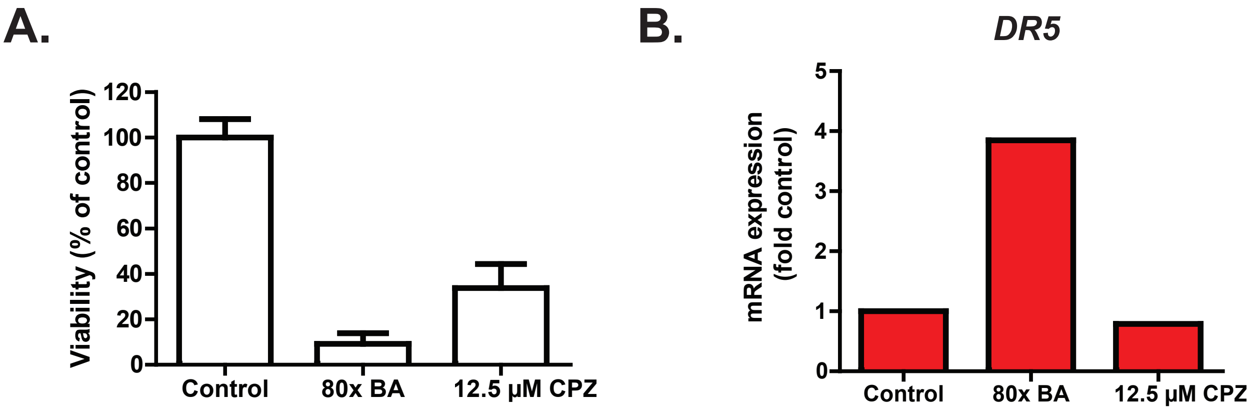

Supplementary figure S3. DR5 expression profile of PHH spheroids exposed to toxic concentrations of chlorpromazine or bile acids.

PHH spheroids were repeatedly exposed to a toxic concentration of chlorpromazine or BAs. After 8 days, viability was assessed by measuring cellular ATP content (A) and expression levels of DR5 (B) were evaluated by RT-qPCR and normalized to the expression of the housekeeping gene GAPDH.
